# Women's perceptions of changes following the intervention on the prevention of mistreatment in gynecology and obstetrics in Guinea

**DOI:** 10.3389/frhs.2026.1860891

**Published:** 2026-09-09

**Authors:** Alpha Oumar Sall, Mamadou Dioulde Balde, Sadan Camara, Kaba Saran Keita, Moussa Camara, Boubacar Alpha Diallo, Madeleine Toure, Aissatou Diallo, Fanta Barry, Tiany Sidibe

**Affiliations:** 1 Centre for Reproductive Health Research of Guinea (CERREGUI), Conakry, Guinea; 2 Chair of Gynecology and Obstetrics, Gamal Abdel Nasser University, Conakry, Guinea

**Keywords:** Guinea, gynecology, intervention, mistreatment, obstetrics, perceptions, women

## Abstract

**Context:**

The mistreatment of women in gynecology and obstetrics is practiced in all regions of the world. Studies conducted in Guinea have reported compelling evidence of this practice in gynecology and obstetrics services within health facilities. A prevention and response programme against this mistreatment was initiated and implemented in different regions of the country. The intervention for the prevention and struggle against this mistreatment in gynecology and obstetrics focused on raising awareness among women in the community through community health workers and community relays who received training for this purpose.

**Purpose:**

This study aims to determine the changes adopted after the implementation of the intervention by comparing the perceptions of women in the community before and after the intervention.

**Methods:**

This was a qualitative study conducted before and after the intervention to assess the changes perceived by women in the community. A purposive sample of 30 women before and 30 after the intervention was used. Community health workers and community relays, who also live and work within the community, identified and selected participants who met the criteria. Data were collected through in-depth individual interviews (IDIs) with 60 women of reproductive age in the community and analyzed using a thematic approach. Community awareness-raising for the prevention of mistreatment was carried out by community health workers and community relays.

**Results:**

The women in the community mostly reported a reduction in the frequency of mistreatment in gynecology and obstetrics, especially physical violence. However, according to some women, verbal violence and other forms of mistreatment persist.

**Conclusion:**

This intervention has shown signs of improvement in preventing and combating physical violence, but structural and relational forms of violence persist.

## 1 Introduction

The mistreatment of women during gynecological and obstetric care constitutes a fundamental violation of Human Rights and a major obstacle to improving maternal health globally (1). Some women who attend gynecological and obstetric health services experience mistreatment. This practice is widespread in several countries and encompasses many types (1, 2). Health providers’ knowledge and attitudes toward this maternity-related mistreatment often remain limited, and they rarely acknowledge its existence to their clients (3, 4). This mistreatment of women undermines the efforts made over the past few decades to provide women with quality care in health facilities (5). It also leads to mistrust in client-provider relationships, which can significantly discourage the use of maternal and reproductive health services (1). In Africa, previous studies have documented the prevalence of these practices (6). The evidence suggests that this mistreatment is not merely the result of individual behaviors, but is rooted in systemic failures: lack of training, overwork, inadequate infrastructure, and the sociocultural normalization of some forms of violence (7). In Guinea, the practice of mistreatment in obstetric care units exists in several forms (8–11). However, with the aim of giving birth to a healthy child, a woman must benefit from a clinically and psychologically safe environment, accompanied by ongoing practical and emotional support from a caregiver as well as caring and qualified healthcare professionals (12). To ensure such an environment, the adoption of respectful maternal care is recommended, guaranteeing all women access to healthcare that preserves their dignity, privacy, and confidentiality, while ensuring the absence of harm and mistreatment. This care should also enable women to make informed choices and receive ongoing support during labor and delivery (12). Based on this reality, Guinea initiated and implemented a project aimed at preventing and fighting against mistreatment of women during the provision of gynecological and obstetric healthcare services in health facilities. This project comprised a pre -intervention and a post-intervention phase. The pre-intervention phase was carried out in the form of a situational analysis, with the results published in scientific journals (9–11). The intervention itself focused on training healthcare providers and community health workers to prevent women's mistreatment in gynecological and obstetric services. The other phase of the intervention focused on raising awareness among women in the community, conducted by trained community health workers (ASCs) and community relays (RECOs), to guarantee women receive dignified treatment that respects their rights (5). However, despite growing recognition of the problem, there is a lack of data on the effectiveness of interventions aimed at reducing these practices in obstetrics and gynecology services, particularly those incorporating a community component. Most research has focused on documenting the problem or training healthcare providers, without assessing how these changes are perceived by the women concerned. By giving a voice to women in the community, this research makes a crucial contribution to the scientific literature. The objective of this qualitative study is to highlight the changes reported by women in the community after the implementation of the intervention.

## 2 Methods

### 2.1 Study type

This was a qualitative study conducted before and after the intervention to assess the changes perceived by women in the community. Pre-intervention data were collected in 2024 during a baseline situational analysis, while post-intervention data were collected in 2025.

### 2.2 Description of the intervention

The first phase consisted of training healthcare professionals at the intervention sites. The training focused on recognizing abuse of women in gynecology and obstetrics, describing its manifestations, and the role of healthcare professionals in combating these practices. Particular emphasis was placed on the adoption of respectful care and women's rights in sexual and reproductive health. It began with a training-of-trainers workshop on preventing and combating mistreatment of women in gynecology and obstetrics in February 2024. Two managers from each intervention site participated in the workshop. Subsequently, the workshop participants trained, at each site, 10 health care providers working at the regional/prefectural hospital, 12 health care providers from urban and rural health centers, and 60 health workers and community health workers. The intervention thus trained a total of 110 healthcare providers and 300 health workers and community health workers from 25 healthcare facilities across the 5 study sites.

The second phase focused on healthcare providers educating female clients in the gynecology and obstetrics departments of urban and rural hospitals and health centers at the intervention sites on best practices in sexual and maternal healthcare within health care facilities.

The third phase consisted of raising awareness among women of childbearing age within the community. Led by community health workers and community relays, this awareness campaign was conducted on an individual, face-to-face basis. Each week, every community health worker and community relay conducted an individual, face-to-face awareness session with a woman of childbearing age at the various intervention sites.

### 2.3 Context

#### 2.3.1 General context

Guinea is situated on the west coast of Africa and covers a total area of 245,857 km^2^. The resident population of Guinea is estimated at 17, 521,167 inhabitants in 2025, with a population density of 71 inhabitants per km^2^. The gender distribution shows a predominance of females, with 51.8% women compared to 48.2% men. The 8,078 majority (61.3%) of the Guinean population lives in rural areas. Conakry concentrates slightly more than half 8,179 (50.3%) of the country's urban population (13). The proportion of women who gave birth in a health facility was 53% compared to 47% who gave birth at home in 2018 (14). The Guinean health system is organized according to a three-tier pyramid: primary, secondary, and tertiary. The primary level, the first point of contact for the population, comprises 407 health centers overseeing 1,640 health posts, supported by community health workers and community relays involved in awareness campaigns, primary malaria care, and vaccination activities. The secondary level consists of 25 prefectural hospitals, eight regional hospitals, and five community health centers in Conakry, while the tertiary level includes three national hospitals concentrated in the capital, all operating within a decentralized system at the district health level (15).

#### 2.3.2 Specific context

The study was conducted in five prefectures (Boké, Labé, Dabola, Faranah, and Guéckédou) distributed across all the natural regions of the country. These prefectures were selected because of their cultural, geographic, and socioeconomic diversity, allowing for the documentation of different types of vulnerabilities affecting women of reproductive age. In each health district, the study covered health facilities, including a regional or prefectural hospital, an urban health center, and a rural health center, as well as the women in the communities served by these health facilities.

### 2.4 Study population

The study involved women in the community who were sensitized by health workers and community health workers attached to the selected health facilities at each site: the regional or prefectural hospital, as well as one (1) urban health center and one (1) rural health center, were chosen randomly.

### 2.5 Sampling and sample

#### 2.5.1 Sampling

A purposive sample of 30 women before and 30 after the intervention was used. In each prefecture, 6 women of childbearing age who had received gynecological or obstetric health services during the month prior to data collection were interviewed at each phase. Community health workers and community relays, who also live and work within the community, identified and selected participants who met the criteria. Data saturation was determined during the data collection process. Debriefing meetings were held daily between data collectors and local supervisors. These meetings reviewed the information gathered for each topic. The central supervision team also met with local supervisors to discuss the information collected at the various sites. Data collection was halted when it was determined that it was no longer yielding any new information.

#### 2.5.2 Sample size

A preliminary estimate of the sample size was established at 60 women of childbearing age. In each phase, 30 women were interviewed. These women were divided into two age groups: 18–24 years and 25–49 years. At each site, 6 women were recruited, 3 in urban areas and 3 in rural areas per phase. When this was reached and it was agreed no new information was noted, recruitment ceased.

### 2.6 Data collection

The data were collected by women with proven experience in qualitative data collection. They were doctors, midwives, and senior public health technicians. The staff members were assigned to the data collection sites based on their ability to speak the main local languages. Each interview was conducted by a team of two fieldworkers. While one facilitated the interview, the other took notes. The interviews took place within the community in a location that offered quiet, privacy, and security.

The individual interview guides, developed for data collection in the field, were translated into the local languages of the data collection sites (Susu, Pular, Maninka, and Kissi) and then pre-tested. The interviews were conducted in each participant's native language. The data was recorded on voice recorders, transferred as audio files to computers, then transcribed into French and saved as Word files.

During the data collection training, data collectors were instructed to avoid social desirability bias and compliance bias on the part of the participants. The data collection tools, the data collection process, and the data analysis were designed with confirmation bias and the observer effect on the part of the researcher in mind.

Indeed, to ensure the reliability and validity of the results, the data collection and analysis process included a logbook maintained by each data collector to promote self-reflection; daily meetings were held to cross-check data from different data collectors; and in-depth interview guides with open-ended questions were used to explore themes without prompting specific responses. Finally, cross-coding and peer debriefing were employed to minimize the impact of individual biases.

### 2.7 Data saturation

Data saturation was determined during the data collection process. Debriefing meetings were held daily between data collectors and local supervisors. These meetings reviewed the information collected for each theme. The central supervision team also met with local supervisors to discuss the information collected at the various sites. Data collection was halted when it was determined that it was no longer yielding any new information. Thematic saturation was also observed during the data analysis process. No codes required additional information.

### 2.8 Conceptual framework

**Figure 1:**
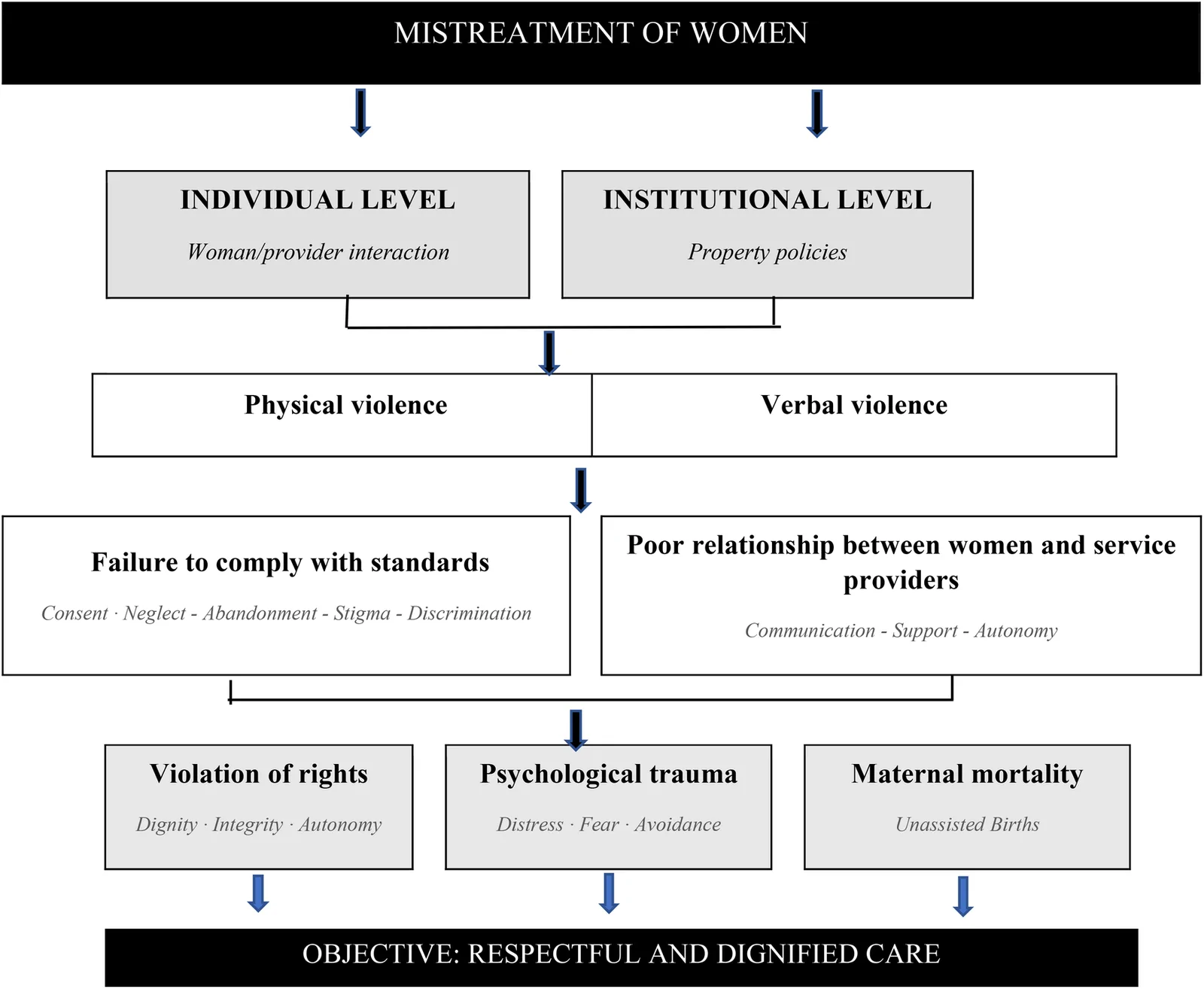
Conceptual framework for mistreatment of women in the context of gynecological and obstetric care.

This study adopted the conceptual framework below, which is an adaptation of the typology of mistreatment of women during childbirth in health facilities (Figure 1).

### 2.9 Data analysis

The interviews were fully transcribed into French before analysis using NVivo 14. The data were analyzed using a thematic approach, as described by Braun and Clark (16). It followed a two-phase inductive approach: (1) open coding of all transcripts by six researchers working independently. Each researcher used two interviews to develop codes. These codes were compared, harmonized, and adopted for coding the entire dataset. (2) Based on the research question, the coding process helped analyze ideas to identify similarities, differences, and patterns. The codes were recorded in a notebook in a reflective and iterative manner. The codes were grouped into themes to explore questions regarding the participants’ lived experiences, behaviors, and practices during their gynecological and obstetric care. Disagreements were resolved through discussion among the researchers or, in cases of persistent disagreement, by consulting another researcher. The credibility of the results was strengthened through internal data triangulation (systematic comparison of interviews) and theoretical triangulation (comparison with data from the literature). This study adheres to the COREQ (COnsolidated criteria for REporting Qualitative research) guidelines for qualitative studies.

## 3 Results

### 3.1 Socio-demographic characteristics of participants

One-quarter of the 30 participants (26.6%) in the situational analysis were in the 20–24 age group. More than four out of five participants were married (80%), and nearly half (43.3%) had never attended school. One-third of the women were homemakers (36.7%). In contrast to this first wave, among the 30 women interviewed after the intervention, nearly half were in the 20–24 age group (43.3%). More than one-third of the women had completed primary school (36.7%). Two-thirds of the participants were married (66.7%), and saleswomen accounted for one-third of these women (33.3%). (Table A1).

### 3.2 Practices and frequency of mistreatment of women

Most women interviewed reported a reduction in mistreatment of women. However, they demonstrate the persistence of these practices, despite the implementation of the intervention. The quotes below are telling:

“So, that's why some women prefer to give birth at home regardless of the cost, because currently,1there are too many young people at the hospital. Even to give you a proper examination, they can't do it, and when you leave, you have stomach pains, and they perform vaginal exams brutally. It's because of all that some women prefer to give birth at home” (IDI_Bok_CR_18–24 years old, after intervention).

“That's what I was explaining: they insult women and call them grimy. Sometimes they make sick women wait when there are many sick people, and sometimes, even if there aren't any sick people waiting, they focus on their phones” (IDI_Far_CR_18–24 years old, after intervention).

### 3.3 Types of mistreatments

The types of mistreatments observed are as varied in the pre-intervention phase as in the post-intervention phase. They include physical, verbal, and other forms of violence. Cases of physical violence persist and are illustrated by this housewife's account:

“When you go to the hospital to give birth, if the healthcare providers ask you to lie down for examination and they find that you are about to give birth even though your legs are pressed together, the midwives might abruptly spread them apart for you, or they might hit you hard while telling you to spread your legs. They might also insult you or whisper in your ear..” (IDI_Lab_CU02_30 years old, before intervention).

A woman living in an urban area also testifies in these terms, adding instances of verbal violence:

“When I go to the hospital, sometimes the way the staff work is not satisfactory. For example, if it's for childbirth, you're in pain, and when they look at you once and decide you're not ready, they'll send a trainee to look at you; if you speak, they yell at you” (IDI_Dab_CU2_36 years old, before intervention).

Some women interviewed after the procedure also emphasized other various forms of mistreatment such as lack of confidentiality, attempted extortion of money from customers, delays in care, long waits, unavailability of healthcare providers, and abandonment.

- Lack of Confidentiality

In most cases, women are treated with respect for confidentiality, according to the results of interviews conducted after the intervention. However, there are cases in which the relatives of the women interviewed experienced a lack of confidentiality. One woman living in an urban area stated:

“Yes, one of my sisters was undergoing fertility treatment at the maternity ward of our hospital here. The staff at that maternity ward can't keep quiet when a woman confides in them; they try to keep the patients’ secrets. The main problem with the mistreatment is the lack of confidentiality and discretion towards the patients.” (IDI_Gue_CU01_40 years old, after intervention).

Another woman living in a rural area reported an experience that illustrates the lack of confidentiality.

“…for example, once, I went to the hospital to accompany one of my friends, but there was a woman there, the woman left. Then the doctor came out, telling his friends that the woman who just left had infections, but it smelled bad, while we are patients. So, in front of us, he shouldn't say that because it's abnormal.” (IDI_Far_CR_26 years old, after intervention).

**•** Neglect and abandonment

Neglect and abandonment are among the most frequently listed negative behaviors of healthcare providers by women. A divorced woman and mother of six describes the neglect women still experience as follows:

“You can leave home to come to the hospital for treatment, but the healthcare workers aren't quick, there are too many people, you end up sitting there for a long time, having left your children behind and not having prepared anything to eat. You come to the hospital because you’re not well and you’re forced to wait. You come in the morning and leave in the evening; sometimes you don't get home until around 6 p.m. This is one of the difficulties we encounter when we come to healthcare facilities…”(IDI_Lab_CU01_39 years old, after intervention).

**•** Attempted extortion of money from customers

A few difficulties related to attempts to extort of money from customers were reported by women in both urban and rural areas:

“They can have difficulties such as money problems. If you go to the hospital and you don't have money, you’ll suffer; you won't get the necessary products. If you don't have money, these difficulties arise.” (IDI_Bok_CR_02_43 years old, after intervention)

“Yes, I can explain others; when you go, they might also offer you a sum of money, but if it turns out you don't have enough, all of this contributes to the difficulties there. Another difficulty is the humiliation of women. If you try to ask a staff member for guidance on what to do, they yell at you. All of this is part of the difficulties.” (IDI_Bok_CU_01_40 years old, after intervention).

A woman living in the city elaborated on the abandonment of women by healthcare providers, linked to the problem of extortion, stating:

“Other healthcare workers may ask you for money you can't afford; you won't be given medication or treatment without money. Some women can die for lack of money.” (IDI Gue_CU01_40 years old, after intervention).

### 3.4 Perpetrators of mistreatment

The main perpetrators of mistreatment are the same in both the pre- and post-intervention data. Indeed, trainees and midwives were the perpetrators of mistreatment reported. For example, one woman reported the following in the pre-intervention phase:

“Yes, it's true, some of them are trainees. During my last delivery, I encountered these cases. Sometimes they yell, whip, scold, and make you leave, telling you, ‘When you're conscious, we'll take care of you." (IDI Lab_CU_01_25 years old, before intervention).

A woman living in a rural area also stated in the post-intervention phase:

“Yes. But it's the trainees who do this a lot. Because when a woman comes for a consultation at the hospital, they yell at you, they insult you from the trainees. In my opinion, when you see that the trainees do this, it's because they aren't employed. The money they earn there isn't much. I think that's it.” (IDI Far_CR_49 years old, after intervention).

### 3.5 Victims’ reactions

Both in the pre-intervention and post-intervention phases, the women interviewed reported no reaction from themselves or their loved ones if they were victims of mistreatment in the gynecology and obstetrics departments. This is illustrated by the statements below from a participant in the pre-intervention phase and another in the post-intervention phase.

- Pre-intervention woman:
  “She (the victim) did nothing wrong; she kept the faith because she went to get treatment. So, she kept the faith so she could get the medication.” (IDI_Dab_CU_01_FA_28ans, before intervention).
- Post-intervention women:
  “No! I didn't complain because even if you speak up, it won't do any good” (IDI_Lab_CU02_25–49 years old, after intervention).
  “Well, if I were experiencing that kind of mistreatment during my pregnancy and I truly had no other choice, I would submit to them until I reached the end of my labor. In short, let's say that after all, I'm complaining about them. Because when I'm suffering, I can't act, and they're the ones with the power, so I have to submit to them until my problem is resolved” (IDI Far_CU02_18–24 years old, after intervention).

### 3.6 Factors associated with the occurrence of mistreatment of women in gynecology and obstetrics

The elements cited in the pre-intervention analysis were also reported in the post-intervention evaluation, with the exception of the need for training for healthcare providers. Persistent challenges included the lack of experience among healthcare providers, insufficient financial resources, the need for supplies and equipment, and the absence of sanctions against healthcare providers. While the need for initial training was not questioned, some women interviewed emphasized the lack of experience among young healthcare workers, as reported by this woman from a rural area:

“When you go to the hospital, there are also young healthcare providers who don't know their job, because if you know your job, you won't just leave the patients and go. But if you know your job, you’ll follow up with your patients and help them. Others don't know their job at all.” Otherwise, if you see a woman who is suffering, who is having difficulties, you must help her. (IDI_Bok_CR_25–49 years old, after intervention).

The extortion of clients was mentioned in the pre- and post-intervention phases. It is reportedly linked to the low wages of some healthcare workers, particularly trainees.

“Here, there are trainees who aren't paid. So, when women come, they take advantage to ask for money, because otherwise, they don't get paid. All of this happens here.” (IDI.Dab_CR02-25–49 years old, after intervention).

### 3.7 Suggestions for preventing mistreatment

The main suggestions made to prevent mistreatment are identical in both phases of the study: availability of medications and equipment, and awareness-raising among healthcare providers and women in the community.

“What you need to do to prevent or stop mistreatment in healthcare facilities is, first of all, to advise midwives to take good care of women who come for consultations; we are like them and we are considered part of their family.” (IDI_Gue_CR_18–24 years old, after intervention)

“I advise all women to go to the hospital for checkup because when they go to the hospital, they will be cured and well treated. So, I also ask the government to properly train doctors for the well-being of all.” (IDI_Dab-CU1_25–49 years old, before intervention).

In addition, strict supervision should be implemented in gynecology and obstetrics departments.

“Furthermore, the authorities must regularly inspect healthcare facilities to see what is happening in hospitals and how providers are treating their patients.” (IDI_Lab_CU02_25–49 years old, after intervention).

## 4 Discussion

The study revealed progress in preventing mistreatment of women in gynecology and obstetrics services in Guinea. However, further efforts are needed to reduce verbal violence, neglect, and attempts to extort money from women practices in health facilities. Trainee health workers are still identified as the primary perpetrators of gynecological and obstetric violence. Our study shows that physical violence experienced in gynecology and obstetrics services was rarely reported after the intervention, contrary to the results of the pre-intervention phase (9–11). It is thus necessary to build on the findings and conclusions of this study to strengthen the training of healthcare professionals particularly trainees in providing respectful care to women in gynecology and obstetrics departments. This reflects a positive change in the attitudes of healthcare providers who received training on preventing and fighting women mistreatment in gynecology and obstetrics, as well as the success of community-based awareness campaigns conducted by community health workers and community relays. Similar results were reported by Arora et al. (2021) in Maharashtra, India, who indicated the feasibility and positive influence of a multi-component intervention, including training for healthcare providers, to improve the preparedness of healthcare professionals to respond to violence against women in resource-limited settings (17). Similarly, Mathonnet and Arvis (2023) also reported that a training-based intervention in Togo led to a 33% increase in healthcare providers’ knowledge of obstetric violence observed in their daily lives, and when focusing specifically on the section regarding physical violence or discrimination; particularly among midwives; their knowledge increased by 80%, according to the study's findings. Like our study, this one proved to be effective primarily in addressing physical violence rather than other forms of violence (18). Women living in the intervention sites reported experiencing verbal violence in health facilities after the intervention. To address this, Jolivet et al. (2021) noted that health providers must communicate effectively using understandable, respectful, and polite language; greet and address clients by name; and provide verbal support and encouragement. Positive and supportive nonverbal communication with clients is another important behavior that demonstrates the right to dignity and respect. Finally, respect and dignity are manifested through sensitivity and empathy toward women and partners experiencing loss or bereavement (19). However, in a multicenter study conducted before and after the intervention, Onyango-Ouma et al. (2001) highlighted that before the intervention, younger health professionals were often impolite toward older clients, while after the intervention, they were more respectful (20). Women reported neglect and long waiting times despite interventions that included training healthcare providers and raising awareness among women in the community. It is important to note, however, that this aspect of women mistreatment is primarily linked to the organization of healthcare facilities. Referred to as health system constraints, Bohren (2015) describes them as a lack of resources, such as infrastructure to guarantee the right to privacy, supplies to ensure that standards of care are met, and staff to ensure that providers are not excessively stressed and can effectively meet the needs of each woman and newborn (1). Yadav et al. (2022) also confirmed that obstacles hindering the delivery of respectful care by healthcare providers include a lack of resources and staff, communication problems, the absence of policies, high workloads, and language barriers (21). Jardim (2018) also gives an explanation commonly put forward by health professionals in the attempt to “justify” the violent scenario of obstetric care, elements such as work overload, lack of human resources, physical and mental exhaustion of health workers, precarious conditions of care delivery, and lack of adequate infrastructure in health facilities (4).

## 5 Strengths and limitations of the study

This is the first study to address mistreatment in gynecology services, whereas previous data were limited to obstetrics, particularly childbirth. It was conducted in most healthcare facilities across the Guinean healthcare system, thus involving healthcare providers with diverse profiles as well as women of reproductive age.

The women of childbearing age (18 to 49 years) included in the study were divided primarily into two age groups: 18–24 years and 25–49 years. However, the pre- and post-groups exhibit sociodemographic differences (age distribution, educational level, marital status) that may influence perceptions of mistreatment and limit the direct comparability of results between the two periods.

## 6 Implications for practice and research

The results of this study showed signs of improvement in women's perceptions of mistreatment in gynecology and obstetrics in Guinea. In practical terms, healthcare providers should step up efforts to deliver healthcare that respects women's rights across various healthcare facilities. At the institutional level, it is necessary to promote the prevention of mistreatment of women in gynecology and obstetrics through training for healthcare providers, raising awareness among women, and equipping healthcare facilities. In terms of research, controlled or quasi-experimental studies would be needed to confirm the causal effectiveness of the intervention.

## 7 Conclusion

This study showed that an intervention focused on training healthcare professionals and raising awareness among women of childbearing age in the community about best practices in sexual and reproductive health appears to contribute to perceived improvements in the prevention and response to the mistreatment of women in gynecology and obstetrics in Guinea. Controlled or quasi-experimental studies would be needed to confirm the causal effectiveness of the intervention.

These results emphasize the importance of systematically integrating respectful care and the prevention of women mistreatment in gynecology and obstetrics into the initial and continuing training curricula for healthcare providers. Sustaining and expanding this type of intervention, with a monitoring and evaluation component, could guarantee maternal health services that respect women's rights and contribute to reducing maternal and neonatal mortality.

## Data Availability

The original contributions presented in the study are included in the article/Supplementary Material, further inquiries can be directed to the corresponding author.

## Appendix

**Table:** TABLE A1 Sociodemographic characteristics of women in the community before and after the intervention.

Variables	IDI			
	Before Intervention		After Intervention	
	(Situational Analysis)		(Post-Experimentation Research)	
	N	%	N	%
Age (years)				
15–19	5	16.7	2	6.7
20–24	8	26.6	13	43.3
25–29	8	26.6	1	3.3
30–34	2	6.7	3	10.0
35 et plus	7	23.3	11	36.7
Occupation				
Housewife	11	36.7	8	26.6
Dressmaker	6	20.0	5	16.7
Saleswoman	8	26.6	10	33.3
Pupil/Student	3	10.0	5	16.7
Civil servants	2	6.7	2	6.7
Education level				
None	13	43.3	6	20.0
Primary	5	16.7	11	36.7
Secondary	5	16.7	4	13.3
Vocational	4	13.3	8	26.7
University	3	10.0	1	3.3
Marital status				
Single	3	10.0	6	20.0
Married	24	80.0	20	66.7
Divorced	1	3.3	3	10.0
Widowed	2	6.7	1	3.3
Number of children				
0 children	3	10.0	4	13.3
1 to 2 children	13	43.3	13	43.4
3 to 4 children	7	23.3	7	23.3
5 or more children	7	23.3	6	20.0
Living environment				
Rural	10	33.3	10	33.3
Urban	20	66.7	20	66.7
